# A Proposed Neurological Interpretation of Language Evolution

**DOI:** 10.1155/2015/872487

**Published:** 2015-06-01

**Authors:** Alfredo Ardila

**Affiliations:** Department of Communication Sciences and Disorders, Florida International University, Miami, FL 33199, USA

## Abstract

Since the very beginning of the aphasia history it has been well established that there are two major aphasic syndromes (Wernicke's-type and Broca's-type aphasia); each one of them is related to the disturbance at a specific linguistic level (lexical/semantic and grammatical) and associated with a particular brain damage localization (temporal and frontal-subcortical). It is proposed that three stages in language evolution could be distinguished: (a) primitive communication systems similar to those observed in other animals, including nonhuman primates; (b) initial communication systems using sound combinations (lexicon) but without relationships among the elements (grammar); and (c) advanced communication systems including word-combinations (grammar). It is proposed that grammar probably originated from the internal representation of actions, resulting in the creation of verbs; this is an ability that depends on the so-called Broca's area and related brain networks. It is suggested that grammar is the basic ability for the development of so-called metacognitive executive functions. It is concluded that while the lexical/semantic language system (vocabulary) probably appeared during human evolution long before the contemporary man (*Homo sapiens sapiens*), the grammatical language historically represents a recent acquisition and is correlated with the development of complex cognition (metacognitive executive functions).

## 1. Introduction

Diverse disciplines have contributed to advancing our understanding on the origins and evolution of language: linguistics, neuroanatomy, archeology, comparative psychology, and genetics [1–27]. As a matter of fact, the origins and evolution of human language represent particularly complex and intriguing questions. According to Christiansen and Kirby [28] understanding language evolution represents the hardest problem in contemporary science.

This paper does not attempt to further review and discuss the origins and evolution of language, but rather to relate the origins of human language, with contemporary cognitive neurosciences, particularly with the aphasia area. Given the complexity of the topic, evidence not only from aphasia, but also from brain evolution theory, linguistics, genetics, anthropology, and psychology will be examined to further the central idea taken from aphasia literature; that is, there exist two language systems supported by different brain circuits, probably appearing at different historical moments in time.

In spite of the potentially significant contribution that aphasia knowledge can make towards understanding the origin of human language, limited interest has been observed in using the aphasia model to approach language evolution [29, 30]. Code [31] has clearly stated that some aspects of aphasic symptomatology may represent fossilized clues to the emergence of human language and hence aphasia analysis can positively contribute to comprehending the evolution of language. He further proposes that the evolution of lexical speech automatisms (such as language clichés, overused social expressions, automatic speech, and the like) to agrammatism (a type of language pattern frequently found in severe nonfluent aphasia) might also provide useful insights into the early evolution of language. Code [32] suggests that “commonly occurring lexical speech automatisms may reflect substages of development from single repeated expletive and syntactically primitive pronoun + modal/aux constructions, forming a bridge to a protosyntax stage, to agrammatism, thus bridging a gap between protolanguage and full syntax” (page 143). As a matter of fact, he considers that lexical speech automatisms could represent some of the earliest utterances appearing in human evolution. This type of analysis presented by Code [31, 32] clearly illustrates that aphasia can indeed significantly advance our understanding about human language evolution.

In this paper, initially, some fundamental observations about language disturbances in the case of brain pathology (aphasia) are reviewed. It is emphasized that throughout the history of aphasia it has been accepted that there are two fundamental types of aphasia syndromes. Although these two fundamental types of aphasia syndromes have been named in different ways (e.g., motor/sensory; anterior/posterior; nonfluent/fluent; etc.) (see Table 1), each one of them is associated with the disturbance of one of two different language elements (lexicon and grammar). This is a basic distinction that has to be considered when analyzing language evolution. Lexicon (vocabulary) and grammar (morphosyntax) are supported by different neural networks and can be independently impaired in cases of brain damage; hence, they present quite different cerebral organization. Interestingly, vocabulary and morphosyntax acquisition are also based on different learning types (declarative and procedural learning; [34–36]) and probably emerged at quite different historical moments. Integrating this basic distinction in an interpretation about historical language evolution can significantly advance our insight of language evolution.

It should be noted that Bickerton [5] has emphasized that there are two most central issues in language evolution: (a) how did symbolic units (words or manual signs) evolve? (b) How did syntax evolve? He considers that symbolic units (i.e., lexicon) and syntax (i.e., grammar) are the only real novelties in human communication systems and are therefore the most important points to approach in a theory on language evolution. He further explicitly points out “there is no reason to believe that the emergence of the two was either simultaneous or due to similar causes, and some good reasons for supposing the contrary” (page 512). To support this argument, he refers to Chomsky's [37] distinction between the conceptual and the computational aspects of language. According to this proposal the conceptual elements (conceptual structure, lexical instantiation) must be significantly older than any computational mechanism (grammar). However, “symbolic units” can be understood in different ways, and depending on how they are defined, it could be argued that even they exist in animal communication systems [38, 39]. We should assume that Bickerton refers specifically to the symbolic units of human language. Bickerton [5] points out that simple logic indicates that symbolic units (lexicon) must exist before any procedure to link these units (grammar). That is, lexicon phylogenetically should have appeared long before grammar. This is exactly the point of view that will be argued in this paper.

## 2. There are Two Fundamental Aphasia Syndromes

Aphasia is generally defined as the loss or impairment of language caused by brain damage [40]. Different subtypes of aphasia syndromes are often mentioned in neurology and cognitive neurosciences, including Broca's aphasia, Wernicke's aphasia, conduction aphasia, amnesic aphasia, and transcortical aphasia [41–46]. The exact number of aphasia subtypes depends on the particular classification, but usually between four and seven different aphasic syndromes are mentioned. Seemingly, this suggested diversity of aphasic syndromes has obscured the major and basic distinction in aphasia: there are only two major aphasic syndromes (see Table 1) [47].

Assuming that there is a significant number of aphasic disturbances (usually between four and seven; sometimes even more) may result in the implicit hypothesis that human language includes diverse discrete abilities, such as phoneme recognition, lexical memory, morphosyntax, repetition ability, and naming. Each one of these abilities would consequently be associated with the activity of a particular cerebral area. These diverse aphasia syndromes (such as Broca's aphasia, conduction aphasia, Wernicke's aphasia, anomic aphasia, and transcortical sensory aphasia) are further regarded as the disturbance of a specific language ability: phoneme recognition, morphosyntax, repetition, and so forth. In consequence, it can be conjectured that human language is based on seven (some times more) language abilities.

It is important to emphasize that since the very beginning of the aphasia history, it has been clearly pointed out that there are only two basic aphasic syndromes (see Table 1), named in different ways, but roughly corresponding to Wernicke's-type aphasia and Broca's-type aphasia [40–43, 46–56]. This has been most basic idea throughout the history since the very beginning of aphasia analysis. For instance, Hippocrates (~400 BC) in his pioneer analysis of language impairments associated with brain damage clearly referred to two different types of language disturbances:* aphonos*, “without voice,” and* anaudos*, “without hearing.” Antonio Guaneiro during the XV century reported two aphasic patients: one with a fluent paraphasic speech and the other one with a nonfluent speech. Later, in 1825 Bouillaud (French physician) distinguished two different types of language pathology: one had an articulatory basis, and the other pathology was amnesic in nature. In 1843, Jacques Lordat (a professor of anatomy and physiology at Montpellier in France) proposed a similar dichotomy; he described the inability to produce words, referred to as* verbal asynergy*, and the disturbance in the ability to recall words, referred to as* verbal amnesia *[57, 58]. This distinction between two major language disturbances represents the most basic information in aphasia: “*aphasia is not a single unified language disturbance, but two rather different (even opposite) clinical syndromes” *(page 29) [48].

These two fundamental aphasic syndromes are associated with a disturbance at the level of the language elements (lexical/semantic) in Wernicke's aphasia or at the level of the association between the language elements (morphosyntactic/grammatical) in Broca's aphasia. It has been further observed that these two basic dimensions of language (lexical/semantic and grammatical) are related to two basic linguistic operations:* selecting* (that means the language as a paradigm) (“paradigm” in linguistics is usually understood as a set of linguistic items that form mutually exclusive choices in particular syntactic roles) and* sequencing* (that means language as syntagm) [59–61].

**Figure 1:**
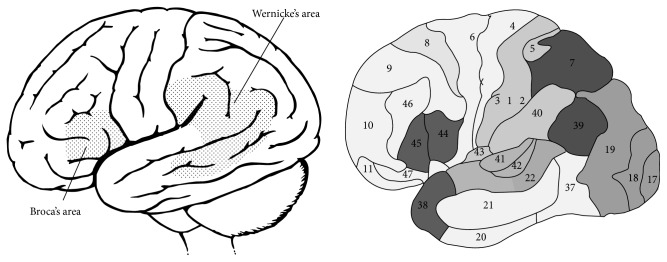
Traditionally it has been accepted that there are two major areas involved in language: frontal Broca's area (BA44 and probably BA45) and temporal Wernicke's area (BA22, 21, and 37, although BA39 is also frequently included.).

Jakobson [62] suggested that aphasia can involve one of two potential types of languages defects: language can be impaired as a paradigm (in Wernicke's aphasia) or as a syntagm (in Broca's aphasia) (Figure 1). In other words, in aphasia, the lexicon (vocabulary) and the grammar (morphosyntax) can be independently impaired [48, 63] and, hence, lexicon and grammar depend not only on different brain areas, but also on different cerebral networks. From a purely linguistic perspective Chomsky [64, 65] has clearly illustrated that the lexical/semantic system is independent from the grammatical system; that is, a sentence can be grammatically correct but semantically empty. Ardila [33, 48, 63, 66] has explicitly proposed that there are two different language systems in the brain: lexical/semantic and grammatical system, supported by different brain areas (temporal and frontal) in the left hemisphere, developed at different ages during child's language acquisition, and appearing at different historical moments during human evolution. The mechanisms for learning are also different for both language systems: the lexical/semantic knowledge is based on a particular type of memory known as “declarative memory” (facts and knowledge we are aware of), whereas grammatical knowledge corresponds to a “procedural memory” (memory about how to perform a particular action) [34–36]. A disturbance in each one of these types of memory is associated with a specific subtype of language impairment: lexical/semantic disorder impairment in Wernicke's aphasia and grammatical disorder in Broca's aphasia. Mental lexicon depends on temporal lobe substrate of declarative memory, whereas mental grammar (rule-governed combination of lexical items into complex representations) depends on specific frontal, basal ganglia, parietal and cerebellar structures [67].

Jakobson is usually considered as a pioneer of the structural analysis of language. Regardless of the fact that most ideas that he proposed have been integrated in contemporary linguistics, his interpretations have not been free of critics (e.g., [68]). Currently, there is a poststructuralism movement that has not only advanced previous theories, but formulated new proposals in the structural analysis of language (e.g., [69, 70]).

### 2.1. The Lexical/Semantic Disorder

The selection disorder observed in Wernicke's aphasia limits the patient's ability to select words (impairment of the paradigmatic axis of the language), that is, to select the elements of the vocabulary. Word selection and word-use errors are observed; and there are some different potential errors: (a) nouns simply become inaccessible; sometimes they are replaced by more general words (for instance, instead of* dog* the patient says* animal*); (b) there are difficulties in selecting between semantically related words (*cat, dog, horse, fox*, etc.), and semantic substitutions (so-called “semantic paraphasias”) are observed. (c) Frequently, these patients fill out their discourse with so-called circumlocutions (“to go around in speech”); for instance, the clock is referred to as “*the instrument used to know the time.*”

Luria [61] reanalyzed the proposal presented by Jakobson [59–62] and suggested that the selection (paradigmatic) disorder could potentially be observed at three different language levels; the disturbance in each one of these levels would be associated with a specific aphasic syndrome: (a) disturbance in phoneme selection that is observed in the so-called acoustic agnosic aphasia (a subtype of Wernicke's aphasia according to Luria), (b) disturbance in word selection associated with a different subtype of Wernicke's aphasia referred to by Luria as an acoustic amnesic aphasia, and, finally, (c) error in selecting the word association, that is, the semantics of the words, correlated with the so-called amnesic aphasia. Similarly, the sequencing (contiguity) disorder can potentially be found at two different levels: (a) when sequencing words in a sentence, as is observed in Broca's aphasia (designated by Luria as kinetic motor aphasia), or (b) in sequencing sentences in discourse, found in so-called transcortical motor aphasia (named by Luria as dynamic aphasia). It is interesting to keep in mind that different subtypes of Wernicke's aphasia are frequently distinguished (e.g., [40, 71]). For Luria, so-called acoustic agnosic aphasia, acoustic amnesic aphasia, and amnesic aphasia are simply subtypes of the aphasia syndrome usually referred to as Wernicke's (or sensory) aphasia.

**Figure 2:**
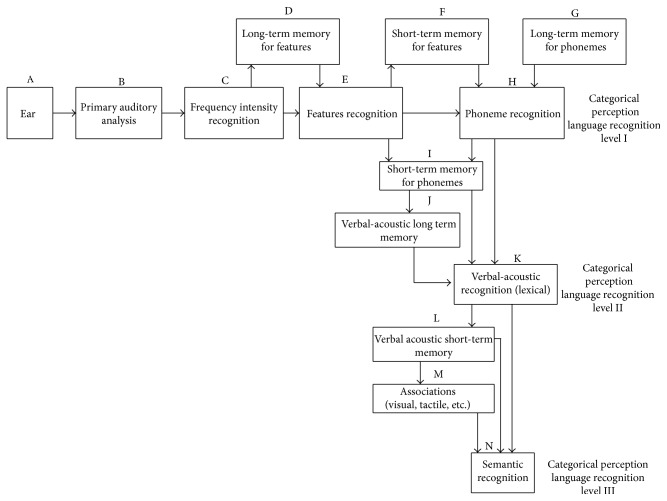
Diagram model for language recognition proposed by Ardila [33]. Three levels of language recognition potentially impaired in Wernicke-type aphasia can be distinguished: phonemic (categorical perception level I), lexical (categorical perception level II), and semantic (categorical perception level III). Three different subsyndromes can be found: phonemic discrimination defects (acoustic-agnosic or Wernicke aphasia type I), verbal-acoustic memory defects (acoustic amnesic or Wernicke aphasia type II), and semantic association defects (amnesic, nominal, or Extrasylvian sensory aphasia).

As a matter of fact, in Wernicke's aphasia different language deficits can be found: the lexical knowledge (vocabulary) may be decreased resulting in difficulties in understanding spoken language. Sometimes (particularly in cases of damage close to the primary auditory area) phoneme discrimination defects are also found. Furthermore, words can lack a precise meaning, and semantic disturbances are observed, associated with left temporal-occipital pathology. So, it can be conjectured that three different defects account for the language impairments found in Wernicke's aphasia: (a) phoneme discrimination impairments; (b) language memory abnormalities; and (c) association defects between words and meanings.  Figure 2 presents the model proposed by Ardila [33] in an attempt to integrate the language abnormalities found in Wernicke's aphasia. According to this model, there are three different levels of language recognition that can potentially be impaired in Wernicke's aphasia; they are the phonemic, the lexical, and the semantic level. The impairment in each one will result in a particular subtype of Wernicke's aphasia.

Neuroimaging studies clearly reinforce the heterogeneous role of the left temporal lobe in processing auditory information. For example, Leaver and Rauschecker [72] analyzed how the brain processes complex sounds, like voices or musical instrument sounds. Using functional magnetic resonance imaging these authors were able to identify category-selective responses in the anterior superior temporal regions, consisting of clusters selective for musical instrument sounds and for human speech. An additional subregion was found that was particularly selective for the acoustic-phonetic content of speech. Regions along the superior temporal plane closer to primary auditory cortex were not selective for stimulus category, responding instead to specific acoustic features embedded in natural sounds.

It has to be emphasized that, in Wernicke's aphasia, the language abnormality is situated at the level of the language elements (words, vocabulary). Phoneme and word selection can be impaired, but language morphosyntax (grammar) is not impaired. Nonetheless, sometimes patients with Wernicke's aphasia tend to overuse the grammatical elements, resulting in a phenomenon usually referred to as paragrammatism [73].

It has been observed that nouns are apparently associated with an organized pattern of cerebral activity. According to contemporary clinical and functional studies, the knowledge of different semantic categories (e.g., animals, musical instruments, and body parts) may be separately represented in the brain [74, 75]. It is well known that anomia (difficulties for finding names) may differently impair naming body parts, naming external objects, and naming colors [76]. Moreover, the naming defect can be limited to a particular semantic category (for instance, naming living things, tools, and geographical places) [77–80]; the naming defect can be so specific as to refer just to “medical terms” [81]. It has been consequently suggested that there is a kind of “brain mapping” of the word memories, associated with different semantic categories [71].

Departing from studies with monkeys it has been proposed that there is a dual-stream (anterior and posterior) in the auditory cortex [82]. However, close homologies between human and monkey cortex have been found. Consequently, two different systems can be distinguished in Wernicke's area: dorsal and ventral. The left superior temporal gyrus (ventral stream) supports auditory word-form recognition, whereas superior temporal/inferior parietal lobules (dorsal stream) support functions of “inner speech” [83].

### 2.2. The Grammatical Disorder

Disturbances in grammar are observed in Broca's aphasia. Jakobson [59–61], departing from a purely linguistic perspective, suggested that in Broca's aphasia there is a basic defect in the sequencing process. It has been well established that as a matter of fact, Broca's aphasia includes two different abnormalities: (a) a motor production defect characterized by decreased fluency, abnormalities in the speech kinetic melodies, articulation slowness, and so forth, referred to as* apraxia of speech*, and (b) a disturbance in the use of grammar usually known as* agrammatism* [40, 46, 51, 84]. It has been conjectured that if both impairments (apraxia of speech and agrammatism) are simultaneously found, they simply represent two apparent manifestations of a fundamental defect [85]. It has been suggested that the “inability to sequence expressive elements” observed at the phonological/articulatory level (resulting in so-called apraxia of speech) or at the purely linguistic level (resulting the so-called agrammatism) could be such a fundamental defect.

During recent years a significant interest in cognitive neurosciences for understanding the specific role of Broca's area has been observed. There has been the implicit assumption that understanding Broca's area is fundamental for understanding human cognition. As mentioned above, it can be assumed that Broca's area is not really specialized in producing speech, but rather in a fundamental neural process responsible not only for speech movements, but also for grammar use. Noteworthy, deafmute individuals (consequently not using speech) frequently present difficulties in understanding and using language grammar [86].

Meta-analyses of functional neuroimaging studies (particularly fMRI and PET) have indicated that grammatical processing is clearly related to the left inferior frontal gyrus, including Brodmann's areas 44 and 45, corresponding to Broca's area [87, 88]. In an illustrative experimental study, Petersson et al. [89] investigated a group of subjects on a grammaticality classification task; the participants had been previously exposed to well-formed consonant strings generated from an artificial regular grammar. The aim of the study was to find whether brain regions related to language processing overlap with the brain regions activated by the grammaticality classification task used in this research. The authors observed that artificial grammaticality violations activated Broca's region in all participants, emphasizing the involvement of this brain region in grammar knowledge and use.

Noteworthy, some authors have suggested that left inferior frontal gyrus is involved only complex syntactic processing (demanding increased cognitive control and working memory) (e.g., [90–92]).

## 3. Three Different Stages in Human Language Evolution

Departing for the mentioned linguistic and neurological observations (i.e., there are two different language systems in the brain and there are two fundamental types of aphasia) and Bickerton's [5] suggestion that symbolic units (lexical/semantic system) and syntax (grammatical system) are the only real novelties in human communication system, which probably emerged at different historical moments, three different stages in language evolution could be proposed:

- Primitive communication systems: they use some sounds but may also include other types of information, such as gestures and grunts. These communication systems obviously correspond to the communication systems found in other animals, including nonhuman primates.
- Initial language systems using combined sounds to form words but without a relationship among the words (grammar): that means language as lexical/semantic system but not yet as a grammatical system. This type of language is similar to the holophrastic period observed in children at the beginning of language development, around the age 12–18 months [86].
- Advanced communication systems using word-combinations (grammar): that means language as grammatical system. At advanced ages in children, not just an increase in the vocabulary is observed, but also the beginning of grammar; around the age of 24–30 months children begin to combine words into simple sentences. Initially, utterances including two words without connecting elements; later, grammatical connectors appear [93].

I am suggesting that human language initially emerged as a collection of significant combination of sounds (words; the paradigmatic axis of the language, according to Jakobson [57–60]) and only later evolved toward a system of relations between these words (the paradigmatic axis of the language, according to Jakobson [59–62]). This point of view is congruent with Bickerton's [5] proposal about language evolution stages: lexicon phylogenetically should appear long before grammar.

### 3.1. First Stage: Primitive Communication Systems

Animals use different communication systems, based in different sensory modalities: visual, auditory, and even olfactory. Without question, initial human language was similar to the communication systems observed in other hominid primates, such as chimpanzees, orangutans, gorillas, and gibbons.

It is known that chimpanzees use a diversity of gestures (including facial expressions) to communicate [94]. In addition, they have a limited repertoire of vocalizations (they produce about 12 different vocalizations) that can be used for communication purposes with other chimps. Observations of nonhuman primates communication strategies have been collected in different conditions, including natural environments and also laboratory groups in human-controlled environments [95]. Interestingly, chimpanzees can learn some artificial languages (such as using tokens) and close to about 200 “words” (symbols).

It has been found that a chimp's ability to learn complex communication systems has not been particularly successful. K. J. Hayes and C. Hayes [96] trained the chimp Vicki in a human environment. Her ability to learn a human language was limited to four different words in several years! Similar experiments have been carried out with other chimps and gorillas with similarly limited success [97–99].

Noteworthy, whereas nonhuman primates can learn a relatively high amount of “words” (e.g., Kanzi learned to use some 200 symbols) they have significant difficulties in learning to combine these “words” (to use grammar); that means it is not evident that nonhuman primates can learn the language syntax [100, 101].

The crucial question in language evolution is how to move from the language as a collection of words to a grammatical language. For humans, creating new words does not seem specially complicated. As a matter of fact, it has been proposed that in human history certain mechanism could have been used to create new words (for instance, words can be created departing from onomatopoeias or emotional expressions) [102]. However, considering the limited amount of vocalizations found in nonhuman primates, it can be concluded that these primates have a limited ability for the creation of new significant elements in communication (“words”).

### 3.2. Moving to a Human Language

The origin of human language has been for centuries a particularly controversial topic. During the 19th century, different hypotheses were presented to account for the emergence of human language; however, these proposals did not include the origins of grammar but were restricted to the origins of the lexical/semantic system (vocabulary) [102, 103]. Some of these hypotheses are the following:

1. Language began as imitations of natural sounds. In other words, onomatopoeias (onomatopoeia is a word that phonetically reproduces the source of the sound that it refers to) represent a basic mechanism for the creation of new words. Indeed, this is a very important mechanism to create new words, and, as a matter of fact, every human language contains an important amount of words that originally were onomatopoeias (e.g., hiccup, zoom, bang, beep, moo, and splash). Some words that currently do not look like onomatopoeias originally were onomatopoeias; for instance, the word “barbarian” is derived from Greek* barbaros* “foreign, strange, ignorant,” from the root* barbar* (onomatopoeia of unintelligible speech of foreigners) [104].
2. Gestures are at the origin of language, and body movement preceded language. Oral language represents the use of oral gestures that began in imitation of hand gestures that were already in use for communication. Recently, different authors [6–8, 105] have argued that gestures represent the most important element in creating human language.
3. Language began with interjections, emotive cries, and emotional expressions. In fact, emotional communication continues playing a significant role in contemporary human communication under certain particular conditions, such as highly emotional situations [103].
4. Language began with the easiest syllables attached to the most significant objects (e.g., /ma/). Because the easiest syllables are the same for every child anywhere worldwide, some early words are quite similar across different languages (e.g., /mama/) [103].
5. Language arose from rhythmic chants and vocalisms uttered by people engaged in communal labor [103].
6. It has been observed that there is a certain correspondence between language sounds (phonemes) and meanings; that is, words maintain some relationship with the meaning. Small, sharp, high things tend to have words with high front vowels in many languages (e.g., /i/in “little”), while big, round, low things tend to include back vowels (e.g., /a/in “large”). This relationship is often referred to as “phonetic symbolism” [103, 106] and has been demonstrated in a diversity of languages (for a review, see [107]). Phonetic symbolism simply refers to the notion that phonemes can convey meaning on their own, apart from their configuration in words [108].
7. It has been also suggested that language comes out of play, laughter, cooing, courtship, emotional mutterings, and the like [102].
8. Considering that there is a need for interpersonal contact, language may have begun as sounds to signal both identity (here I am!) and belonging (I'm with you!); this is known as contact theory [102].

These hypotheses are not contradictory, and indeed all these mechanisms may have contributed to the creation of new words. Nonetheless, these hypotheses attempt to explain how the language vocabulary was created; hence, how language evolved from the first (primitive communication systems) to the second stage (language as a lexical/semantic system). They do not include any explanation for the development of what could be considered as more characteristic of human language: language grammar. Indeed, these mechanisms for creating new words are still used in contemporary languages. For instance, onomatopoeias are still a significant strategy for the creation of new words (the name of the game ping-pong is clearly departing from onomatopoeia).

### 3.3. The Role of Vocalizations (Noises, Grunts) in Human Communication

Regardless of the significant amount of vocalizations (noises, grunts) used in everyday human communication, little mention to them is found. As a matter of fact, vocalizations represent a basic communication strategy in different nonhuman primates including chimpanzees, and without question, they have continued playing a communication function throughout human history. People in everyday life frequently use a diversity of noises (vocalizations) to say “yes,” “no,” to express different emotions, to make emphasis, and so forth. These vocalizations are close to interjections, and sometimes become real interjections (e.g., “ooph!”).

### 3.4. Second Stage: Initial Communication Systems

Bickerton [4] proposed that a protolanguage must have preceded the full-fledged syntax of today's discourse. Some echoes of this initial protolanguage can be found in, (a) in pidgin languages (pidgin is a simplified language that develops for communication among people that do not have a common language), (b) in the initial words that children develop, (c) in the symbols used by trained chimpanzees in artificial conditions, and finally (d) in the syntax-free utterances of some children who do not learn to speak at the normal age. Bickerton [109] considers that such a protolanguage existed already in the earliest* Homo* (about 2.3 to 2.4 million years ago) and was developed due to the pressure of the behavioral adaptations faced by* Homo habilis *(2.3 to 1.4 million years ago).

What made up these original words? Again, the analogy with the child's initial vocabulary can be taken. (a) They were simple and easy to produce from the articulatory point of view; probably, they included those phonemes regarded as “universal” phonemes (i.e., they are found across all the world languages, such as /m/, /a/). (b) The phoneme sequence was also simple (consonant-vowel); these simple syllables may have been produced in a repeated way (e.g.,* mamama*). (c) They obviously were “nouns” (real objects), something that is directly experienced.

To create articulated words requires the progressive development of a series of articulatory oppositions [13]. According to Jakobson [110] the most basic one is the opposition between vowels and consonants. The second most important articulatory opposition is between oral and nasal phonemes. But the production of these oppositions requires some anatomical adaptations in the phonatory (vocal folds, larynx) and articulatory (tongue, lips, palate, etc.) systems.

Human articulatory ability is partially due to the specific position and also configuration of the larynx. Interestingly, the human larynx descends during infancy and the early adolescent years; it is assumed that this descent significantly contributes to the anatomical requirements for speech articulation. Although this developmental phenomenon is frequently considered to be unique to humans, Nishimura [111, 112] demonstrated that indeed chimpanzees' larynx is similar and also descends during infancy, as observed in humans. Probably, the descent is associated with developmental changes of the swallowing mechanisms. But most important, it also contributes morphologically to an increased independence between the processes of phonation and articulation for speech production.

Interestingly, laryngeal descent in nonhumans is not accompanied by descent of the hyoid [113]. In humans, lowered larynx increases the vocal tract length, increases the potential vocal sounds repertoire, reduces the frequency of resonances, and makes sounds louder [114, 115].

Different researchers have proposed that there are some universal language characteristics, found across world languages and even in some attempts to reconstruct extinct languages that have been presented [116–118], for instance, to reconstruct the Indo-European language (a central language for most languages in spoken in Europe, the Middle East, and India) [115, 119–122] that disappeared over 10,000 years ago. Similarly, some proposals about the initial human vocabulary have also been presented; so, Swadesh [123] refers to some universal words existing across different languages (kind of “basic vocabulary”). According to the Swadesh's “basic vocabulary” [26, 124], the following categories are found across different languages and may represent the initial word categories: (a) grammatical words (e.g., I/me), (b) quantifiers (e.g., all), (c) adjectives (e.g., big), (d) human distinctions (e.g., person), (e) animals (e.g., fish), (f) highly frequent elements (e.g., tree), (g) body parts (e.g., hair), (h) actions (e.g., drink), (i) natural phenomena (e.g., sun), and (j) colors (e.g., red).

### 3.5. Third Stage: Advanced Communication Systems

The evolution of grammar (“grammar” or morphosyntax refers to the rules governing the use of language and includes “morphology,” the study of word formation, and “syntax,” the study of how words are combined into larger units such as phrases and sentences) represents the most complex and poorly understood question in language evolution. Noteworthy, human languages, regardless of the diversity in their details, present profound structural similarities in all regions of the world (i.e., there is core syntax or universal grammar) [5], suggesting an original grammar, or at least, some universal principles for expressing ideas resulting from the specific human brain idiosyncratic organization. 

Some proposals have been presented to account for the historical origins of grammar [125, 126]. Klein and Edgar [127] proposed that a mutation in the human species may have occurred about 50,000 years ago, accounting for the full human language (i.e., grammatical language). The rationale behind Klein's claim refers to the fact that human culture significantly accelerated shortly after the date, resulting in a rapid increase in the amount of produced elements, including the first symbolic artifacts (statuettes, cave-paintings, etc.). As will be mentioned below, this acceleration in culture development may have been related to the development of so-called “metacognitive executive functions” (such as planning, abstracting, problem solving ability, and temporality of behavior) [128]. Metacognitive executive functions are strongly linked to the internal representation of actions, to the use of verbs, and to the development of a grammatical language [48, 63]. To determine the exact date in which this occurred is obviously extremely difficult, but it could be around the date proposed by Klein and Edgar [127] (about 50,000 years) or even later.

So what was the crucial leap for the development of grammar? (i.e., syntagmatic dimension of the language). Bickerton [5] stated this question in a direct and clear way: “the emergence of our own species released a torrent of creativity that is still gathering speed. What caused this difference? Clearly, it is some startling increment in cognition. But what caused cognition to change so dramatically? The emergence of modern syntacticized language is the most plausible, indeed perhaps the only serious contender” (page 520). Here, it will be argued that the modern syntacticized language and the development of metacognitive executive functions (the increased cognition Bickerton refers to) are simply two sides of the same coin.

Grammar begins with the ability to combine two words to create a new higher level unit (a syntagm, two or more linguistic elements that occur sequentially in the chain of speech and have a specific relationship). But how can we pinpoint the particular relationship between these two words? Obviously, the procedure has to be the simplest one, probably similar to the procedure observed during child's language development.

If we have two nouns such as

 -*baby-toy,*

we can suppose that different relations can be established between these two words; but in order to create a simple sentence, a verb indicating an action is required, for instance,* baby likes toy, baby has toy, *and* baby wants toy. *This means that in order to create a syntagmatic relationship between two or more vocabulary words, different word categories have to be distinguished, specifically, nouns (objects) and verbs (actions). As a matter of fact, to create a simple phrase, only two types of elements are indeed required: nouns (corresponding to the so-called nominal phrase) and verbs (corresponding to so-called the verbal phrase). Analyzing language development in children, Brown [129] proposed that most of the utterances when beginning grammar development could be described by a small set of functional relationships between words, such as “agent + action” (baby kiss), “action + object” (pull car), and “agent + object” (daddy ball).

The crucial point in emerging grammar is not just the complexity of the lexical/semantic system, that is, the extension of the vocabulary. What is really important is to have words corresponding to different classes that can be combined to form a higher level unit (syntagm, phrase, and sentence). One of the words has to refer to an object (noun); the other is an action (verb). A sentence is usually regarded as a grammatical unit that is syntactically independent and has a subject that is expressed or understood (as in imperative sentences) and a predicate that contains at least one finite verb [130]; that means a sentence contains a subject (noun) and a verb, indicating that two different word categories are required.

Naming actions have been related to left frontal operculum activation [131]. In cases of brain pathology, the ability to use verbs is impaired simultaneously with the ability to use grammar as observed in cases of damage involving Broca's area [132]. For instance, Ardila and Rosselli [133] reported the case of a 33-year-old woman who presented a selective defect in finding verbs and naming actions after a head injury associated with a left frontal posterior hematoma. Objects, colors, body parts, and qualities were named in a normal way. In this case it was clear that the ability to name objects and name actions was clearly dissociated.

It is important to underline that some authors have argued that lexicon and grammar develop simultaneously in human history; for instance, Tomasello [134] assumes that the origin of language is related to the use of gestures, and indeed grammar is already in the action. Consequently, lexicon does not appear before grammar. This disagreement emphasizes that there are important ongoing debates and competing explanations with regard to the origins of human language.

## 4. Brain Evolution and the Origins of Human Language

### 4.1. Origins of the Lexical/Semantic System

To understand the origins of language, it is crucial to consider the evolution of the brain areas involved in language processing, such as the temporal lobe (lexical/semantic system). It is known that in monkeys, the temporal lobes participate in recognizing the sounds and calls of their own species [135–138]. Hence, the temporal lobe plays a crucial role in auditory communication not only in humans but also in nonhuman primates. However, what is the specific adaptation of the temporal lobe that resulted in a significant advance and increase in complexity of human auditory communication?

Gannon et al. [139] observed that the anatomic pattern and left hemisphere size predominance of the* planum temporale*, a language area of the human brain, are also present in chimpanzees. Consequently, this is not a critical difference between the human and chimpanzee brains. Similarly, anatomical temporal lobe asymmetries (favoring the left hemisphere) are also found in different monkey species [140]. Hopkins and Nir [141] used magnetic resonance images to analyze whether chimpanzees present asymmetries in the* planum temporale* for grey matter volume and surface area. The results indicated that the chimpanzees present leftward asymmetries for both surface area and grey matter volumes. Consequently, It could be suggested that leftward asymmetry of the left temporal auditory association area (Wernicke's area) developed prior to the appearance of contemporary human language and probably even before our divergence between humans and chimpanzees [142].

It has been further suggested that temporal lobe differences between humans and nonhuman primates relate to the temporal lobe volume. Rilling and Seligman [143] studied the temporal lobe volume in several primates including humans. It was found that overall volume, surface area, and white matter volume were significantly larger in humans than predicted by the ape regression lines. This increase in several temporal lobe dimensions may be related to the complexity of the human auditory communication system. It is interesting to note that the temporal lobe directly participates in the recognition of the own species sounds, and the superior temporal gyrus contains neurons that are tuned to species-specific calls [144]. On the other hand, it has been proposed that this significant enlargement of the temporal lobe may have occurred about 200–300 thousand years ago [145] suggesting an increase in complexity in the human communication system around this time. Consequently, it can be conjectured that hominids existing before the contemporary* Homo sapiens* could have developed a certain complex lexical/semantic communication system. For instance, it could be speculated that Neanderthal man (*Homo neanderthalensis *if classified as a different species or* Homo sapiens neanderthalensis *if classified as a subspecies of the* Homo sapiens*) could have had a language relatively complex as a lexical/semantic system. It is worth noting that the FOXP2 gene sequence was found in two male Neanderthals dated about 40,000 years ago [146–148]. This FOXP2 gene has strongly implicated in speech and language development [149–152] (see below) suggesting that the* Homo sapiens neanderthalensis* indeed possessed language somehow similar to contemporary human language. Interestingly,* Homo sapiens neanderthalensis *was mostly right handed in a proportion similar to contemporary man [153] and obviously there is a significant association between handedness and language lateralization in the brain [154, 155].

Falk et al. [156] examined the endocasts of* Australopithecus africanus* and three species of* Paranthropus*. They found that the brain morphology of* Australopithecus africanus* appears more human like than that of* Paranthropus* in terms of overall frontal and temporal lobe shape. This finding is consistent with the hypothesis that* Australopithecus africanus* could have been ancestral to* Homo* and has implications for assessing the early hominid neurological and cognitive evolution [157].

Brain organization of the lexicon (vocabulary) depends on the specific type of association between vocabulary words and perception. When a particular word is associated with own body information (for instance, the word “finger”), brain representation of the lexicon seems associated with a parietal extension; when the word has a visual association (for instance, the word “book”), an occipital extension is found [158], and so forth.

### 4.2. Origins of the Grammatical System

It has been suggested that verbs, grammar, and speech praxis (generated spatiotemporal specifications for skilled purposeful articulatory movements) appeared simultaneously in history [33]. Interestingly, grammar, speech praxis, and the ability to use verbs are simultaneously impaired in cases of Broca's area damage, suggesting a common neural activity. So, the origin of the grammar is directly linked to the ability to use verbs and the ability to produce certain articulatory movements. What has been the evolution of Broca's area and associated networks (responsible for grammar and speech praxis) obviously represents a fundamental question.

Genetic observation has shed some light on the question about the origins of neural systems responsible for language production. During recent years, the study of a family affected with language production difficulties significantly advanced our understanding on the origins of language grammar and speech praxis. This is an English family usually referred to as KE family. For over three generations about half of the family members presented important abnormalities in language development. Speech difficulties were evident and articulation was deficient. This disorder was associated with a mutation in a single autosomal-dominant gene, FOXP2, located in the chromosome 7 [10, 149, 152]. In addition to the speech difficulties, affected members also presented defects in processing words according to grammatical rules, the understanding of more complex sentence structure such as sentences with embedded relative clauses, the ability to form intelligible speech, the ability to move the mouth and face in a way not associated with speaking (relative immobility of the lower face and mouth, particularly the upper lip), and general intellectual limitations.

It was suggested that the core deficit in this complex syndrome was one involving sequential articulation and orofacial praxis [159–162]. Brain abnormalities involving so the cerebral cortex and the subcortical areas were documented. An abnormal gene (SPCH1) in the chromosomal band 7q31 was localized. The genetic mutation or deletion in this region was suggested to be associated with significant impairment of speech and expressive language, including grammar [162].

Enard et al. [10] analyzed the evolution of the gene FOXP2. They emphasized the extremely conservative nature of FOXP2. The authors point out that the mouse FOXP2 differs by just one amino acid from chimpanzee, gorilla, and rhesus monkey. However, human FOXP2 differs from gorilla, chimp, and rhesus macaque by two further amino acids (and thus differs from mouse by three amino acids out of 715). That means that in 75 million years since the divergence of mouse and chimpanzee lineages only one change occurred in FOXP2, whilst in the six million years since the divergence of man and chimpanzee lineages two additional changes have occurred in the human lineage. The authors calculated that the last two mutations might have occurred between 10,000 and 100,000 years ago and speculated that the mutations have been critical for the development of contemporary human speech.

This genetic approach to the origins of language seems particularly important in understanding the appearance and evolution of language in humans [163, 164]. It has been pointed out that FOXP2 could have contributed to the evolution of human speech and language by adapting specific corticobasal ganglia circuits to communication purposes [150]. It has been further suggested that although FOXP2 is expressed in many brain regions and has multiple roles during mammalian development, the evolutionary changes that occurred in the protein in human ancestors specifically affect brain regions that are connected via corticobasal ganglia circuits [165].

So, it seems that the development of a human language grammatical system may be related to some specific genetic mutations occurring relatively recently in history. The development of a grammatical language, no question, had a significant impact in the evolution of human complex cognition.

## 5. Grammar and the Origin of Complex Human Cognition

In contemporary cognitive neurosciences, complex cognition is usually related to so-called executive functions [166–172]. Nonetheless, it is not clear what is the unitary and fundamental factor underlying and unifying executive functions [171, 172]. Ardila [128] has suggested that “action representation” (i.e., internally representing movements or actions) and associated with it “behavior temporality,” may represent the fundamental executive functions factor. He suggested that both might depend upon one single core ability (“sequencing?”) [173].

Action representation is evidently related to the use of verbs, because verbs usually refer to actions (temporal changes); consequently, action representation is required for the development and use of verbs and grammar. Some authors have interpreted the use of time concepts and behavioral temporality in general, as the basic factor accounting to executive functions [174, 175]. Notably, executive functions are directly controlled by the prefrontal cortex; and the prefrontal cortex represents an extension and further increase in complexity of the frontal motor areas involved in action performance [176, 177]. Furthermore, significant motor control abnormalities can be observed in cases of prefrontal damage, including primitive reflexes and perseveration [178, 179].

Different authors have emphasized that complex cognition (such as thought, reasoning, and problem solving) depends on an internalization of actions. Vygotsky [180–182] argued that thought (and so-called “complex psychological processes”) is associated with some “inner speech.” Lieberman [15–17] has suggested that language in particular and cognition in general arise from complex sequences of motor activities.

For Vygotsky [181], the central point is that complex cognition depends on certain mediation (especially, but not only by language). Thinking is regarded as a covert motor activity (i.e., “inner speech”). Lieberman [15–17] has on the other hand proposed that the frontal lobes are implicated in virtually all cognitive activities, whereas posterior cortical regions represent active elements in vocabulary knowledge. Many other authors have presented a similar point of view [183–187]. Some recent research seemingly supports this interpretation [188].

The discovery of the so-called mirror neurons (a neuron which fires both when an animal performs an action and also when the animal observes the same action performed by another animal) [189–193] can significantly contribute to a better understanding of the brain representation of actions (verbs). Mirror neurons were initially described in monkeys [189]; in the human brain, the existence of mirror neurons has been suggested in the premotor cortex and the inferior parietal cortex [194]. It has also been suggested that mirrors neurons exist in Broca's area [192]. The discovery of mirror neurons in Broca's area may have important consequences for understanding brain language organization and language evolution [195–198]. Indeed, mirror neurons could be involved in the internal representation of actions and, hence, in the origins of grammar. Notably, it has been suggested that “inner speech” is related to activity of Broca's area [199, 200].

Finally, as a note of caution, it is important to keep in mind that these two recent discoveries, FOXP2 gene and mirror neurons, do not represent a direct and easy answer to the question of language evolution. Bickerton [5] clearly and overtly criticizes the expectation that these recent discoveries (“mirror neurons” and the FOXP2 gene) will provide easy answers about the evolution of human language. He considers that mirror neurons cannot, even in principle, shed any light on how symbols originated or how syntax originated, the two most basic questions in language evolution. On the other hand, he considers that FOXP2 gene may have something to do with human-ape differences, probably including language; but he emphasizes that until we know exactly which other genes FOXP2 turns on or off, it is premature to claim any specific function and simply incorrect to consider it as a major driving force in language evolution.

## 6. Conclusions

Without question, aphasia analysis can significantly contribute to the understanding of human language evolution. According to contemporary aphasia knowledge, in cases of brain pathology, language can be disturbed in two rather different ways: as a lexical/semantic system (Wernicke-type aphasia) and as a grammatical system (Broca-type aphasia). Both language systems not only depend upon different brain areas (temporal and frontal), but are also supported by different neuroanatomical circuitries. This observation is concurrent with contemporary theories of language and language evolution, distinguishing two major elements in language (e.g., symbolic units and syntax; elements and structure; nouns and verbs).

As it was mentioned before the superior temporal gyrus contains neurons that are tuned to species-specific calls. Human brain and old-world monkeys show a great deal of anatomical similarity [201]. The auditory cortical system is organized into a ventral and a dorsal pathway in both species. The similar role of the ventral auditory pathway in both humans and monkeys in the decoding of spectrally complex sounds—including the perception of speech sounds—has been well established. The dorsal processing stream plays a major role in speech production. This idea is quite similar to the current proposal that there are two different language systems in the brain. The main difference is that anterior and posterior superior temporal cortex are distinguished on the basis of nonhuman primate neuroanatomy (e.g., [202]), which leads to the distinction of ventral and dorsal pathways.

Observations with children's language development and experiments with nonhuman primates demonstrate that language initially appears as a lexical/semantic system. Grammar, on the other hand, is correlated with the ability to use verbs and represent actions. This is an ability that depends on, the so-called Broca's area and related brain circuits. But this ability also depends on, is correlated, and likely appeared simultaneously in human history with the ability to rapidly sequence articulatory movements (speech praxis). Furthermore, language grammar probably represents the departing ability for the development of complex human cognition (executive functions).

## Figures and Tables

**Table 1:** Different names used to refer to the two basic aphasic syndromes.

Receptive	Expressive
Sensory	Motor
Ventral	Dorsal
Fluent	Nonfluent
Wernicke-type	Broca-type
